# TREM2/PLCγ2 signalling in immune cells: function, structural insight, and potential therapeutic modulation

**DOI:** 10.1186/s13024-021-00436-5

**Published:** 2021-04-06

**Authors:** Lorenza Magno, Tom D. Bunney, Emma Mead, Fredrik Svensson, Magda N. Bictash

**Affiliations:** 1 Alzheimer’s Research UK UCL Drug Discovery Institute, University College London, Cruciform Building, Gower Street, London, WC1E 6BT UK; 2 Institute of Structural and Molecular Biology, Division of Biosciences, University College London, Gower Street, London, WC1E 6BT UK; 3 Alzheimer’s Research UK Oxford Drug Discovery Institute, Nuffield Department of Medicine Research Building, University of Oxford, Oxford, OX3 7FZ UK

**Keywords:** Alzheimer’s disease, Signalling, Microglia, Protein networks, Therapeutic intervention, Immune system

## Abstract

The central role of the resident innate immune cells of the brain (microglia) in neurodegeneration has become clear over the past few years largely through genome-wide association studies (GWAS), and has rapidly become an active area of research. However, a mechanistic understanding (gene to function) has lagged behind. That is now beginning to change, as exemplified by a number of recent exciting and important reports that provide insight into the function of two key gene products – TREM2 (Triggering Receptor Expressed On Myeloid Cells 2) and PLCγ2 (Phospholipase C gamma2) – in microglia, and their role in neurodegenerative disorders. In this review we explore and discuss these recent advances and the opportunities that they may provide for the development of new therapies.

## Background

Polymorphisms in microglial genes have been associated with both increased and decreased susceptibility to late onset Alzheimer’s disease (LOAD) [1]. The identification of these coding variants not only provides insights into the biological mechanisms involved in the disease, but it can also inform on new, hypothesis-driven, drug-discovery programs. As such, beyond identification and confirmation of expression, further studies are essential to unravel the effect of the variants on protein function, and subsequent microglial functional phenotypes in specific disease-contexts.

Amongst the polymorphisms linked to LOAD, one such example is provided by the related innate immune genes *TREM2* and *PLCG2*. TREM2 is a transmembrane receptor present on cells of the myeloid lineage including central nervous system (CNS) microglia, bone osteoclasts, alveolar and peritoneal macrophages [2].

A main effector downstream of TREM2, PLCγ2 is an intracellular lipase recruited to the membrane upon activation [3, 4]. PLCγ2 is expressed in cells of the hematopoietic lineage [5].

Several variants in TREM2 have been linked to increased LOAD risk [1, 6]. These have been extensively reviewed elsewhere [7]. Conversely, a protective polymorphism in *PLCG2* (rs72824905: p.Pro522Arg) has been associated with: decreased risk of LOAD (odds ratio = 0.68; minor allele frequency cases = 0.0059, controls = 0.0093 [1]), Dementia with Lewy Bodies and Frontotemporal Dementia, slowed disease progression, which correlated with a reduction of pTau_181_ and tTau in cerebrospinal fluid (CSF) [8], and finally promotion of healthy ageing [1, 8, 9]. Moreover, the protective PLCγ2 variant seems to counteract the harmful effect of the *APOE* ε4 allele, as shown for a cognitively healthy centenarian carrying both the homozygous *APOE* ε4 and the *PLCG2* protective polymorphism [9]. Additional haplotypes around *PLCG2* have been recently found to be associated with AD [10–12]. However, the effects of *TREM2* and *PLCG2* polymorphisms might depend on the type of brain disease, stage of neurodegeneration [13] and on gene-dosage, or the level of enzymatic activity produced. Thus, a better understanding of these pathways in immune function, and in specific disease-context, is required to properly design therapeutic strategies.

In the CNS, TREM2 and PLCγ2 are enriched in microglia where they exert an important role in supporting a variety of activities such as lipid sensing, and phagocytosis. TREM2 is required for the conversion of microglia from a homeostatic to a disease activated state [14, 15], which potentially confers neuroprotection by enhancing microglial function, including an up-regulation of genes implicated in migration [16], phagocytosis [17], survival [18] and lipid metabolism [19]. Sensing membrane damage, phagocytosis and clearance of debris represent key microglial responses to neurodegeneration. Additionally, proper regulation of lipid metabolism is essential to support these functions [20]. Of note, restoration of myeloid cell bioenergetics was shown to reverse cognitive decline in ageing [21]. Evidence from these studies, together with the genetic link to LOAD, places these regulatory genes at the core of the microglial “defence mechanisms” against dementia.

In the next sections we aim to 1) review the current knowledge around the TREM2/PLCγ2 pathways in immune cells, 2) offer new insights into the structure and function of these proteins and their variants in disease, and 3) discuss the implications for the development of new therapies for neurodegeneration.

## Main text

### The role of TREM2 and PLCG2 in immune cell biology

#### TREM2/PLCγ2 function in peripheral and CNS immune cells

TREM2 and PLCγ2 function has until recently been more thoroughly investigated in peripheral immune cells than in the CNS, with variants of these genes known to cause diseases such as Nasu-Hakola disease (TREM2) [22], and PLCγ2-associated antibody deficiency (PLAID), or autoinflammation and PLAID (APLAID) [3]. These proteins are critical for the proper maturation, maintenance and function of a range of immune and hematopoietic-lineage cells [5, 23]. TREM2 expression is restricted to myeloid lineage, including dendritic cells and monocytes, as well as parenchymal macrophages residing in various tissues/organs, such as the lungs, bones and liver [4, 23] (https://www.proteinatlas.org/ENSG00000095970-TREM2/celltype [24]). On the other hand, PLCγ2 is more broadly present in myeloid cells [5, 25, 26], neutrophils [25, 27], mast cells [25], NK cells [5, 25], B-cells and platelets [5, 28, 29] (https://www.proteinatlas.org/ENSG00000197943-PLCG2/celltype [24]). In these cells, PLCγ2, downstream of B-cell receptor and Fc receptor signalling, regulates calcium-dependent functions supporting immune and inflammatory responses [5, 28]. See also the paragraph “*PLCG2 variants: the Good, the Bad and the Ugly”* for a review on the consequences of PLCγ2 loss of function (LOF) in peripheral immune cells.

TREM2 and PLCγ2 signalling has been shown to regulate several immune functions in macrophages, promoting both pro- or anti-inflammatory responses, depending on cellular state and influence of the local environment [4]. While ﻿TREM2 activation causes inhibition of the Toll-like-receptor (TLR)-induced proinflammatory cytokine responses in macrophages and dendritic cells [30], PLCγ2 activation leads to an increase in cytoplasmic calcium, that supports the assembly of the pyrin (PYD)-domain-containing protein 3 (NLRP3) inflammasome, and pro-inflammatory cytokine release in human blood monocytes [31], bone-marrow-derived macrophages (BMDM) and dendritic cells [32].

**Fig. 1:**
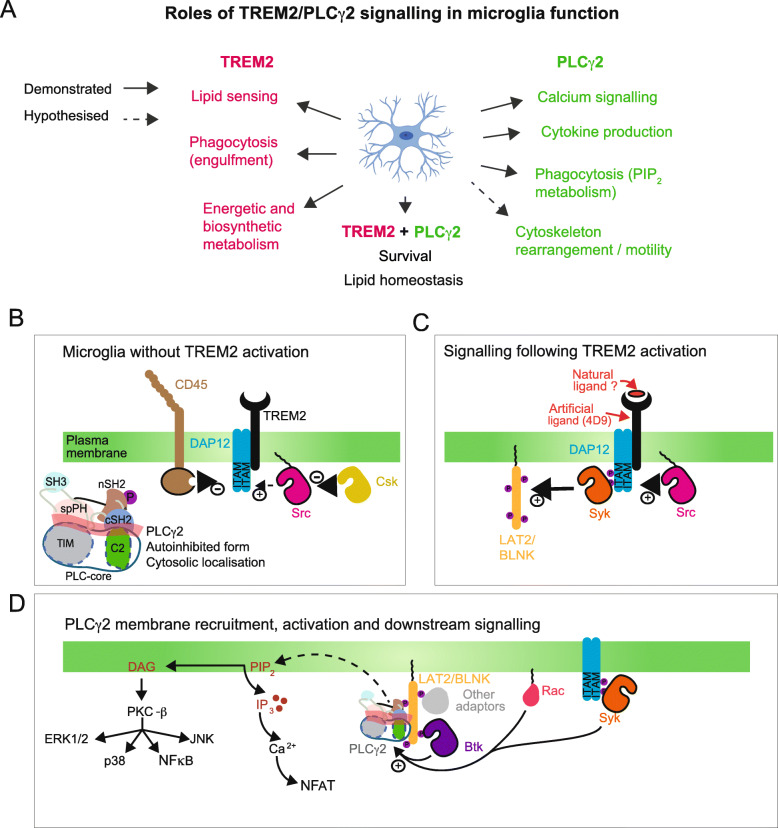
TREM2 and PLCγ2 functions, and signalling networks regulating their activation in microglia. **a** Summary of the microglial physiological functions associated with TREM2 and PLCγ2 activation. **b** A representation of some candidate proteins that are expressed in microglia and maintain TREM2/DAP12 in an inactive state. In absence of activating signals, PLCγ2 is neither recruited to the membrane or activated but instead maintained in its autoinhibited form in the cytoplasm. **c** A possible, simplified, model for the signalling from ligand bound TREM2, through DAP12 and non-receptor kinases (such as Syk and Src) to the adapter protein LAT2/BLNK. Tyrosine phosphorylation on membrane localised adaptors like LAT2 allow the recruitment of signalling proteins like PLCγ2 and other molecules to the vicinity of the plasma membrane where their substrate PIP_2_ is localised. **d** Model for recruitment and activation of PLCγ2 and consequences of PLCγ2 activity in microglial cell signalling, survival and phagocytosis. In addition to interactions with the adaptors that mainly contribute to the recruitment, a number of proteins have been shown to be important for PLCγ2 activation in various immune cells; these include BTK and Syk kinases, mediating tyrosine phosphorylation, and a small-GTPase Rac that could contribute to phosphorylation-independent activation. The relative importance of these inputs in microglia remains to be elucidated

It is possible that similar functions might be exerted by these enzymes in innate immune cell of the CNS. Indeed, investigations in microglia suggested that the TREM2 pathway promotes anti-inflammatory responses [33], while PLCγ2 activation seems to induce pro-inflammatory cytokine secretion [34]. However, more studies are needed to fully address this in microglia. It is worth noting that, in microglia, TREM2 might also signal via other pathways (such as Phosphoinositide 3 Kinase (PI3K)) and PLCγ2 can be activated via other receptors such as Fc. Nevertheless, for the scope of this review we focus on TREM2 and PLCγ2 signalling together, given that: i) protein-protein interaction network analysis [1] and in vitro studies show evidence for interaction of the two proteins in microglia [34]; ii) PLCG2 Knock-out (KO) microglia cells largely phenocopy TREM2 KO cells [34]; iii) in vivo work has validated mRNA co-expression in subcellular domains [26]. Moreover, although their precise function in neurodegeneration is still unclear, both genes display variants that are strongly associated with AD. Microglia functions depending on the TREM2 and PLCγ2 signalling pathways are listed in Fig. 1a.

#### TREM2/PLCγ2 signalling in lipid metabolism

In the CNS, TREM2 interacts with a variety of ligands, but it is most notably a receptor for lipid substrates [17, 19, 48]. TREM2 binds lipids exposed on the surface of apoptotic cells [49, 50], as well as ApoE [51] and lipidated Amyloid beta (Aβ) [52]. In addition to binding lipid ligands, the TREM2 signalling cascade, of which PLCγ2 is a key pathway component, plays an important role in regulating lipid metabolism [19, 34]. TREM2 KO microglia, both in vivo and in human induced pluripotent stem cells (iPSCs), fail to clear cholesterol esters that accumulate as a result of myelin challenge [19]. Furthermore, Piers et al. revealed that AD-associated TREM2 LOF variants, including p.R47H, are defective in lipid binding, which results in the inability of microglia to convert their metabolic state from oxidative phosphorylation to glycolysis [53]. This locked immunometabolic switch may underlie the defective lipid metabolism observed in cells lacking TREM2, or carrying a LOF mutation. Indeed, TREM2 deficiency is known to prevent a conversion of microglia to the disease associated microglial (DAM) state [19], and the transcription of several genes governing metabolism are also modulated in this state [54].

Cholesterol ester aggregates have been found in TREM2 KO and AD-variant human iPSC microglia, and lipid droplets have also been identified in aged/pathological microglia, pointing towards a role for lipid accumulation in AD [20]. In contrast to the observed effect of TREM2 governing lipid metabolism [19, 53], RNA-seq analysis of Lipid Droplet Accumulating Microglia (LDAM) isolated from aged mice revealed there are no changes in TREM2 expression in this cell type [20]. However, phagocytic deficits in LDAM were observed, in line with defective conversion of microglia to the protective DAM phenotype [20], indicating a lack of TREM2-driven phenotypic switch or TREM2 LOF. This could be representative of changes in TREM2 expression or shedding, both of which are altered during ageing and disease [55], thereby reducing TREM2-mediated signalling and subsequent conversion of microglia to DAM [56]. Interestingly, lipid-associated macrophages have recently been identified in mouse and human adipose tissue, where TREM2 expression was found to regulate lipid homeostasis [57]. These cells had a similar transcriptional profile to DAM [57], in agreement with findings from Nugent et al., who demonstrated the importance of TREM2 in the clearance of cholesterol esters [19]. Whilst contradictory, together these studies highlight the pathogenic effect of lipid accumulation, and the importance of lipid metabolism in microglia.

Whilst TREM2 is the receptor for lipid binding, PLCγ2 is implicated as a key signalling node that links TREM2 activation with microglial lipid metabolism, and altered phenotype in KO cells [34]. PLCG2 KO in human iPSC macrophages was shown to inhibit TREM2 signalling, as demonstrated by a reduction in inositol monophosphate (IP_1_) accumulation when compared to wild type (WT) cells. Interestingly, RNA-seq analysis of TREM2 KO and PLCG2 KO human iPSC macrophages revealed that these lines shared the same transcriptional changes, and of note, lipid processing genes, including *LPL, LIPA, APOC1, FABP5* and *PLIN2,* were downregulated in both lines under basal conditions [34]. This correlated with a reduced ability of both lines to clear cholesterol esters, indicating that PLCγ2 is responsible for lipid handling in microglia. Overexpression of the protective PLCγ2 variant, p.P522R, revealed that this mutation enhanced the clearance of cholesterol esters, suggesting that it may act to modulate lipid metabolism and restore microglial functionality. Further analysis of the crosstalk between TREM2 and PLCγ2 showed that PLCγ2 is required for phagocytosis of axonal debris in an axotomy model [34]. Both TREM2 KO and PLCG2 KO iPSC-derived microglia were equivalently deficient in clearing axonal debris, which correlated with an increase in lipid accumulation in these cells. It will be important to see whether additional similarities in phenotype are observed by others to corroborate these findings.

### Microglia signalling networks: linking TREM2 to PLCγ2

#### A parallelism between signalling pathways of immune B-/T-cell receptors and TREM2

Investigating the signalling network that links TREM2 and PLCγ2 will be crucial to understanding the precise roles that these proteins play in physiological microglial function. Currently, we have a rather scant knowledge of PLCγ2 signalling pathways in macrophages and microglia, when compared to PLCγ signalling in B- and T-cells [2, 58]. Nevertheless, there are many parallels amongst these various cells in the proteins involved in linking and regulating cell surface receptors and PLCγ isoforms.

TREM2 function is regulated via interactions and crosstalk with other receptors, including the AD risk hits CD33, and the Ms4A gene-cluster products [23, 59]. A survey of TREM2, its ligands, and potential role in AD have been recently covered elsewhere [23, 60]. The molecular mechanism of how conformational change in TREM2 propagates signals through the membrane to intracellular components is not fully understood. It is known, however, that the TREM2 co-receptor DNAX Activating Protein of 12 kDa (DAP12) becomes phosphorylated on intracellular tyrosine residues. An analogous signal propagation occurs in the T-cell receptor, but even in this well-studied example, with many available structures, not all of the aspects of the molecular details are known [61]. In the context of microglia, the kinase responsible for phosphorylating the Immunoreceptor Tyrosine Activation Motif (ITAM) residues on DAP12 is likely to be Src [62] or a Src-family kinase such as Lyn [63, 64]. Src kinase is a non-receptor tyrosine kinase that is constitutively membrane-localised due to myristoylation at its N-terminus [65]. In many cells, the activation state of Src is held in balance by a number of regulating proteins, which include the kinase Csk and the phosphatase CD45 (Fig. 1b). Src activity is suppressed by phosphorylation on Tyr527 that is catalysed by Csk. The structural basis of this process is well understood [66]. The role of CD45 in blood cells is rather more enigmatic. It is known to be expressed in microglia [67], albeit not to the extent of its expression in T-cells, where it is one of the most abundant proteins in the plasma membrane (PM) [68]. It has been proposed that its role in T-cells is to act as a signalling gatekeeper that prevents T-cell receptor activation by weakly-binding antigens, but promotes activation of strongly-binding antigens. This role could be preserved in microglia and other cells of the innate immune system, but quite possibly its regulatory role could be subtly different.

#### The pathway downstream of TREM2 activation

Although many of the proteins linking TREM2/DAP12 activation to downstream targets have not yet been reported, it is likely that the signalling pathways are analogous to those described in Fc receptor signalling, in as much as Src phosphorylation on the tyrosine residues of DAP12 creates a binding site for the non-receptor spleen tyrosine kinase (Syk). Once recruited to the DAP12 ITAM motif, Syk is phosphorylated by Src on its activation loop and this then initiates an autophosphorylation of other Syk molecules [69] (Fig. 1c). Within B-cells it is known that Syk has a number of targets, which include the adaptor protein B-cell Linker Protein (BLNK) and the Brutons Tyrosine Kinase (BTK); the latter is also an effector of phosphatidylinositol (3,4,5)-triphosphate (PIP_3_), generated by PI3K. Phosphorylated BLNK binds to PLCγ2, and recruited PLCγ2 is phosphorylated and activated by BTK [70].

In BMDMs macrophages, ligation of TREM2 induces the recruitment of the p85 subunit of the PI3K enzyme to the DAP12 complex [71]. ﻿PI3K then converts phosphatidylinositol (4,5)-bisphosphate (PIP_2_) into PIP_3_, which seems to be important for the recruitment of the scaffolding proteins [4]. It is unknown whether this would be an important step leading to activation of PLCγ2.

Within microglia it has been suggested that Linkers for Activation of T- and B-cells (LAT, LAB or LAT2), rather than BLNK, could be phosphorylated by Syk, or that Syk may directly phosphorylate and activate PLCγ2, thus bypassing BTK in these cells [4, 62]. In microglia, it is also unclear to what extent other adaptor proteins are recruited and associated with PLCγ2 at the membrane. In the context of T-cell signalling, it is documented that several adaptor proteins are associated with activated PLCγ1, including LAT, SH2-domain-containing leukocyte protein of 76 kDa (SLP76) and GRB2-related adaptor downstream of Shc (GADS) [72]. An important consideration with regards to PLCγ signalling is that the molecule is localised to the cytoplasm in “resting” cells, and therefore its activation is a two-step process involving recruitment to the membrane compartment, and then removal of autoinhibition (Fig. 1d). In signalling downstream of receptor tyrosine kinases (RTKs), these molecules both recruit and activate PLCγ. In contrast, in T-cells, B-cells and signalling downstream of TREM2 in microglia, these processes are more complex. In microglia, the exact components involved in recruitment or direct activation have not yet been identified.

Another potential component of PLCγ activation, specific to the PLCγ2 isoform and therefore potentially relevant for signalling in microglia, is Rac-driven activation. PLCγ2 activation downstream of Rac is phosphorylation-independent and involves the lipid-modified, GTP-bound form of Rac [73]. Rac is known to be activated downstream of TREM2/DAP12 via Syk and Vav2/3 activation [56]. Although the exact mechanism of PLCγ2 activation driven by Rac is unknown, it is intriguing that the split PH (spPH) domain is now known to be one of the main lynchpins of autoinhibition, and that Rac binds directly to this domain. BTK has been shown to recruit PLCγ2 to the PM, and the activation can occur through Rac in the absence of phosphorylation [74]. Further studies will elucidate if this Rac/PLCγ2 pathway plays an important role in microglial signalling.

Upon activation, PLCγ2 hydrolyses the PM-inner-leaflet-localised lipid, PIP_2_, to produce inositol (1,4,5)-trisphosphate and diacylglycerol (DAG) [75, 76]. Both products of the reaction have roles in intracellular signaling, with IP_3_ production leading to Ca^2+^ release from intracellular stores, and DAG leading to activation of protein kinase C (PKC) isozymes, the NLRP3 inflammasome [31, 34], as well as other proteins [77]. It is not only the increase in the products of PLC activity that should be considered, but also the reduction in localised PIP_2_ that can result from PLC activation. PIP_2_ plays a crucial role in regulating a number of cellular processes or molecules, including actin polymerisation, endocytosis, exocytosis, ion channels and G-protein-coupled receptors [78, 79]. Another consequence of PIP_2_ hydrolysis is the production of protons, which can lead to localised decreases in the juxtamembrane pH [80, 81]. All of these facets would need to be considered when trying to understand the various roles of PLCγ2 in microglial functions.

### Understanding PLCγ2 function and regulation: a structural perspective

**Fig. 2:**
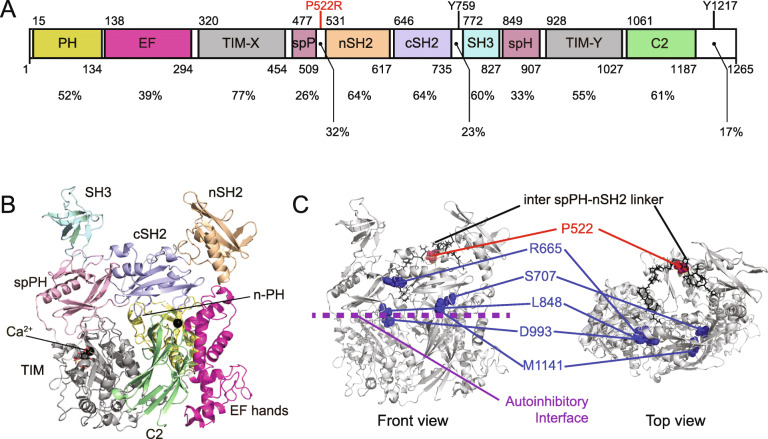
Structural insights relating to PLCγ2 and the AD protective mutation. **a** The domain structure of PLCγ2. The amino acid identities when compared to PLCγ1 for individual domains and some linkers are indicated. Also indicated are the positions of the P522R AD mutation, the Y759 and Y1217 residues potentially involved in enzyme regulation through phosphorylation. **b** Model of the structure of PLCγ2 prepared in the Schrödinger suite (Schrödinger Release 2018–3**:** Prime, Schrödinger, LLC, New York, NY, 2020) using the program Prime to predict loops [85, 86]. The homology model was built from the Uniprot sequence of PLCγ2 (P16885) and the PLCγ1 structure (pdb:6PBC) was used as template. **c** Front and top view of the PLCγ2 structural model with the following features indicated: autoinhibitory interface between the core, catalytic domains and the specific array domains (purple). The location of mutations identified in a range of pathologies (blue). The location of the protective AD mutation on the inter spPH-nSH2 linker (red)

A crystal structure is available for TREM2 [82]. Conversely, there are currently no structures for the full-length PLCγ2 molecule, but we can infer some useful information from the recently-published crystal and cryo-electron microscopy (cryoEM) structures of the closely related PLCγ1 molecule [83, 84]. Both proteins share the same domain structure (Fig. 2a), and have significant amino acid identity: consequently it is possible to derive a homology model of PLCγ2 that includes domain linkers (Fig. 2b). The catalytic capabilities of PLCγ isoforms reside in the core region of the enzyme, which consists of an N-terminal PH domain, EF-hands, TIM-like barrel and a C2 domain. The substrate recognition site and residues involved in inositol-lipid hydrolysis are within the catalytic, TIM-like barrel domain. These core domains are shared by most of the mammalian PI-PLCs. PLCγ isozymes are unique in that their regulatory domains are inserted within linkers in the TIM-like barrel, and consist of a spPH, two SH2 and an SH3 domain. This regulatory region is commonly referred to as the γ-specific array (γSA). The N-terminal SH2 (nSH2) domain is positioned at a distance from the core catalytic domains, and is essentially the principal interaction domain for binding to the phosphotyrosine residues in partner proteins [87]. The partner protein could be a RTK [84, 88] or a phosphotyrosine motif associated with an adaptor protein, such as LAT in T-cells [72], or BLNK in B-cells [89]. The SH3 domain is not visible in the cryoEM structure: this suggests that this domain and its associated linkers are highly flexible in the WT protein. The SH3 domain binds to poly-proline motifs in adaptors and other proteins [90]. The crystal and cryoEM structures reveal how the catalytic and γSA regions interact, thus maintaining PLCγ in an autoinhibited state [83, 84]. Specifically, there are two regions of auto-inhibitory interface. Firstly, contact between the TIM-like barrel and spPH domains and, secondly, contacts between the C2 and cSH2 domains. These inhibitory interactions are released after phosphorylation; selective activation by Rac may occur via a different mechanism. Mutations at either of these interfaces can be sufficient to disrupt the autoinhibition, and lead to an activated PLCγ. The effects of such mutations and variants of these enzymes will be discussed in the next sections.

### The effect of TREM2 and PLCγ2 LOAD-associated variants on microglial function and in the context of amyloid/tau deposition in AD

Computational models place TREM2 and other effectors in the pathway – including Syk and BLNK – in a module associated with microglia responses to Aβ [91]. Several LOAD-associated variants in genes expressed within microglia have been identified [92], which have direct effects on microglial phenotype [93], and can alter the course of the disease. Many of these genes act together in distinct signalling pathways and, of note, TREM2 and PLCγ2 interact in the same cascade (Fig. 1) to modulate microglial migration, phagocytosis, and survival [34, 94].

#### TREM2 and PLCG2 LOAD-associated variants exhibit opposing cellular phenotypes

Several mutations in *TREM2* (at least 27) have now been associated with disease. Homozygous Q33X, Y38C and T66M variants have been identified in patients with Nasu-Hakola disease [44, 47, 95].

AD-associated TREM2 risk variants – including p.H157Y, p.R98W, p.D87N, p.T66M, p.R62H, p.R47H, p.Y38C and p.Q33X [6, 43] – affect TREM2 function by various mechanisms, such as reduced ligand binding (p.R47H [96], p.R62H [97]), reduced cell surface expression (p.T66M [98]), and increased shedding (p.H157Y [45], Table 1). Although the functional effects of these mutations vary, and the impacts on TREM2 signalling require further investigation, these variants seem to predominately result in a degree of TREM2 LOF.

**Table 1:** Summary of the effect of the main LOAD-associated variants on microglia phenotypes and in AD

Variant	Microglia phenotype	AD Clinical phenotype	Further comment
PLCγ2 p.P522R	Improved survival, increased inflammatory responses [35], reduction of cholesterol ester accumulation, Increased phagocytosis [36]	Protection against AD [1], DLB and FTD [9], promotion of healthy ageing [9], slower rate of cognitive decline [8], mitigation of tau pathology in presence of amyloid [8]	Indirect measurements on BMDMs and BV2 cells [35]. Studies carried out on IPSC-derived microglia-like cells [34]
TREM2 p.R47H	Impaired myeloid cell response to plaque in vivo and reduced proliferation [37], increased expression of pro-inflammatory cytokines [38], reduced cell adhesion and altered surface levels observed in iPSC-derived macrophages [39]	Not clear Shown by [40] to increase apraxia, psychiatric and parkinsonian symptoms when compared to non-carriers, but no differences in clinical phenotype observed by [41]	In non-symptomatic carriers, elderly individuals show poor cognitive function [42]). There seems to be too few studies with carriers vs non carriers to draw clear conclusions about the contribution of this variant to disease.
TREM2 p.Q33X	Loss of TREM2 expression [43], no studies into effect on microglia phenotype	Heterozygous carriers show typical AD pathology with brain atrophy [44]	Found in FTD patients [44]
TREM2 p.H157Y	Increased shedding from microglia [45], leading to phagocytosis deficits [46]	Results in an increased risk of Alzheimer’s disease, but the clinical phenotype is not characterised [47]	
TREM2 p.R62H	Impaired phagocytosis of Abeta [17]	Unclear due to rarity of this variant	

The most characterised variant of TREM2 (p.R47H) shows defects in ligand binding [6, 37, 38, 97], which impacts the ability of TREM2 to recognise damage, and induce microglial changes, resulting in a dampened response to pathological insults such as Aβ deposition. As TREM2 elicits its functional effect via activation of PLCγ2, it is not surprising that KO of PLCγ2 produces similar phenotypes and, indeed, PLCG2 KO iPSC microglia and macrophages exhibit reduced signalling, phagocytic deficits, and reduction in lipid metabolism, which are also observed in TREM2 KO iPSC lines [34].

Large-scale GWAS have recently identified several polymorphisms in *PLCG2* associated with AD. A large meta-analysis found a new polymorphism (rs12446759) that confers protection against AD (OR = 0.94) [11]. This is located in a region upstream of the *PLCG2* coding sequence, with unknown effect on expression, or protein function. The same study also identified a mutation in *BLNK* negatively associated with AD (OR = 0.89), with considerable expression-quantitative-trait-loci (eQTL) colocalization in dorso-lateral prefrontal cortex and in microglia [11]. A further polymorphism with unknown function has been identified, which maps to an intron of the *PLCG2* gene and is associated with AD [10].

The best described *PLCG2*AD-associated mutation is rs72824905 [1, 8, 9] (Table 1). In contrast to the TREM2 LOAD variants, which are predominantly LOF and increase the risk of AD, the PLCγ2 variant p.P522R has been identified as a protective gain of function (GOF) mutation [1, 26]. p.P522R has been shown to be a functional hypermorph in assays using human cell lines overexpressing it [26]. In these studies, the activity of the AD-protective variant (measured by P_3/1_ accumulation and intracellular calcium flux) was elevated in comparison to the WT PLCγ2 upon EGF receptor stimulation, but not at basal levels. This is in contrast to the pathogenic mutations that, additionally, show enhanced basal activity when overexpressed in human cells. Furthermore, data in human iPSC-derived macrophages has corroborated this finding, demonstrating that intracellular calcium was elevated following PLCγ2 stimulation with an FcγRII-activating antibody in the P522R variant lines, when compared with the WT [36].

Recent studies in mouse microglia have indicated that basal levels of PIP_2_ may be decreased in cells harbouring the p.P522R variant, suggesting that in the mouse brain the P522R mutation might elicit an increase in basal PLCγ2 function [36]. In agreement with this finding, BMDMs from mice harbouring the p.P522R variant, were found to have increased basal IP_1_ levels when compared to WT [35]. Furthermore, Positron Emission Tomography imaging, using the 18F-FPEA probe to detect Translocator Protein (TSPO) activity, revealed that P522R Knock-In (KI) mice showed an increase in microglial activity when compared to WT littermates [35]. This increased basal activity of the p.P522R variant has only been demonstrated in mouse models to date, and more work is therefore required to determine whether this is also true in human iPSC macrophages from engineered lines, and patient-derived cells.

Whilst TREM2 LOAD variants typically induce a reduction in microglial phagocytosis and survival, the PLCγ2 p.P522R variant causes an increase in these beneficial microglial phenotypes [35, 36]. Studies in mouse BMDMs revealed that cells derived from P522R KI mice had an increased capacity to phagocytose pHrodo-labelled zymosan, in comparison to WT BMDMs [35]. Furthermore, Maguire et al. demonstrated that p.P522R increased Aβ-oligomer clearance when compared with WT expressing microglia, macrophages, and human derived iPSC macrophages [36]. These findings therefore indicate that p.P522R may be protective in inducing clearance of Aβ, however, more work is needed to corroborate these findings. Importantly, the p.P522R variant increases BMDM survival after withdrawal of the macrophage colony-stimulating factor [35]. The p.P522R variant has also been shown to increase lipid metabolism in human iPSC macrophages, with cells harbouring this mutation exhibiting a reduced accumulation of cholesterol esters when compared with WT cells [34]. Whist more work is needed to corroborate these findings, data so far indicates that p.P522R enhances the protective phenotype of microglia.

#### Structural basis for the PLCG2 P522R hypermorphic effect

A number of mutations in *PLCG2* (about 75%), that have been previously implicated in various inflammatory diseases [92–102], actually map to the autoinhibitory interface on the PLCγ2 model structure and can be considered as pathogenic (Fig. 2c). Interestingly, the *PLCG2* mutation that is protective in AD, P522R, is located on the linker in the γSA region that connects the spPH and nSH2 domains (Fig. 2c). This position is clearly distinct from the autoinhibitory interface, and it is linked to a quantitatively lower hypermorphic effect on the activity of PLCγ2 compared to the pathogenic mutations (see the chapter “The spectrum of clinical phenotypes manifested by TREM2 and PLCG2 polymorphisms”).

We can therefore classify mutations in PLCγ2 into two principal groups. The first group includes mutations that cause disease and mainly affect the autoinhibitory interface. These are mostly strongly activating (Fig. 2c). In contrast are the mutations that very mildly activate, the only known example to date is P522R. The phenotypic outcome of these mutations is discussed in the next section.

Another essential aspect is how we can mechanistically explain the mildly-activating phenotype caused by the P522R mutation. As outlined, the mutation is located on a linker between the spPH and nSH2 domains (Fig. 2c). The equivalent linker in PLCγ1 is not visible in either the crystal or cryoEM structures, and that most probably suggests linker flexibility. The introduction of an arginine at this position brings a positive charge in a predominantly electronegative linker. This could have various consequences, including introducing an interaction between this arginine and elements of the autoinhibitory interface, thus causing mild disruption in this region. Alternatively, the positive charge could lead to stabilisation of the activated PLCγ2 at the membrane, due to increased affinity with the predominantly-negative inner leaflet of the PM. This latter possibility means that the phenotype would only be seen when PLCγ2 is activated downstream of receptor signalling. A further possibility is that the mutation may influence the PLCγ2 protein-protein interaction network. This potential re-wiring of the PLCγ2 interactome could imbibe it with properties that are not shared with the WT variant.

#### TREM2 and PLCγ2 LOAD variants in AD: data from mouse models and humans

The TREM2 and PLCγ2 LOAD variants have been shown to affect amyloid and tau deposition in mouse models and patients. Mixed effects were described regarding TREM2 LOF on AD mouse models, potentially depending on the stage of the disease, and the model used. TREM2 deficiency has been reported to result in a higher deposition of amyloid in the hippocampus of 5XFAD mice, and was found to reduce the recruitment of microglia to plaques [16, 50]. Furthermore, studies examining post-mortem tissue from patients with AD revealed that microglia from individuals carrying the *TREM2* R47H mutation failed to surround Aβ plaques, which correlated with an increase in filamentous amyloid deposits and phospho-tau-positive neurites in the brain [16, 103]. A recent report, however, highlights that the variant p.R47H seems to attenuate neurodegeneration in the PS19 model of tauopathy, and a positive modulation of the Trem2 function might lead to increase microgliosis and damage in advanced stages of tauopathy [13]. It is unclear, though, how this relates to humans, as R47H is clearly linked to an increased risk of AD [40, 41]. Additionally, patients carrying this mutation exhibit higher levels of both total tau and phosphorylated tau in CSF compared to non-carriers [104].

The P522R *PLCG2* polymorphism was found to be protective against cognitive decline in patients with high levels of Aβ_1–42_, in comparison to individuals expressing common variants [8]. It will be important to determine whether the p.P522R variant affects amyloid deposition, or tau pathology, in mouse models of AD. From the findings in cell-based models and human disease, it could be hypothesised that p.P522R promotes microglial clearance of Aβ plaques, enhances microglial survival and increases microglial activity, in line with findings in mouse-isolated BMDMs [35]. Despite these advances, the precise role of TREM2 and PLCγ2 in AD pathogenesis still needs to be investigated further. In addition to a role for the TREM2/PLCγ2 signalling pathway in disease, it will also be important to consider independent functions of these two genes that might involve other cascades.

### The spectrum of clinical phenotypes manifested by TREM2 and PLCG2 polymorphisms

**Fig. 3:**
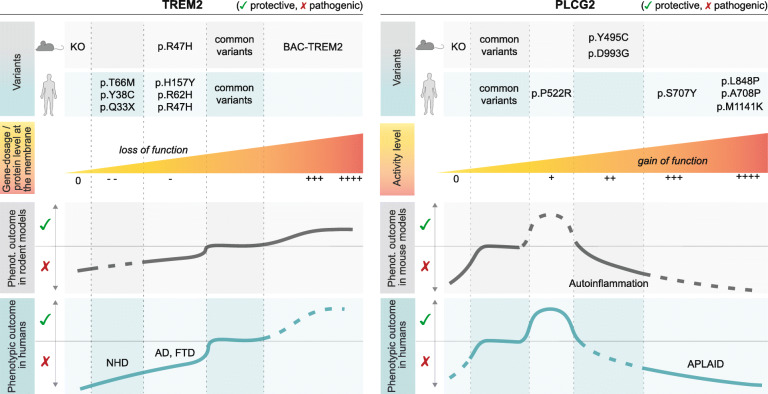
Range of phenotypes of the TREM2 and PLCγ2 variants. Summary of main variants and reported phenotypic consequences in mouse models and humans. Abbreviations: NHD, Nasu-Hakola Disease; AD, Alzheimer’s Disease; FTD, FrontoTempotal Dementia; APLAID, autoinflammation and PLCγ2-associated antibody deficiency

TREM2 and PLCγ2 variants can cause a broad range of effects and disturbances depending on the type of mutation, cell type and organ/system affected. A common theme, however, is the involvement of the immune system and various forms of inflammation. The variety of clinical phenotypes manifested by key *TREM2* and *PLCG2* polymorphisms in humans and mice is summarised in Fig. 3.

#### TREM2 variants

The clinical phenotypes caused by TREM2 variants described above, range from mild to complete LOF (Table 1). TREM2 deficiency leads to adverse phenotypes involving mostly the brain and the bones, and manifesting more severely in humans than mice [42, 105]. Humans with TREM2 deficiency develop the Nasu-Hakola syndrome, characterised by dementia and bone cysts [106], while several Trem2 KO mice show various – sometimes contradictory – phenotypes due to alterations of expression in other genes beyond Trem2 (e.g. Trem1L) in some models [107]. Interestingly, human TREM2 upregulation has been shown to ameliorate neuropathology and memory deficits in 5XFAD and APPswe/PS1dE9 AD mouse models, via microglial molecular reprogramming [108]. It remains to be determined whether increased TREM2 gene dosage results in a potentiation of TREM2 downstream signalling pathway. Most importantly, it would be crucial to demonstrate whether human AD patients would similarly benefit from this increased TREM2 gene dosage and, if so, at which stage of disease (or in which patients) would this effect be most beneficial.

#### PLCG2 variants: the good, the bad and the ugly

In both humans and mice, PLCγ2 loss, as well as strong GOF, mostly results in detrimental and sometimes overlapping phenotypes, as largely described for the peripheral immune system [5, 25].

PLCγ2-deficient mice are viable, but exhibit haemorrhaging in various organs in addition to severe immunodeficiency [5]. PLCγ2 LOF causes impaired Fc-receptor-mediated responses in mast cells, platelets and macrophages [25]. However, the deficits in B-cell development and function are the more pronounced effects described to date. These include completely ablated calcium responses downstream of the B-cell receptor, affecting the ability to respond to stimuli, and reduced antibody production [5]. Although there are no reported cases of humans with PLCγ2 deficiency [101], human iPSC PLCγ2 KO differentiated into microglia-like cells are unable to properly phagocytose, and produce an insufficient response to lipid challenge (see section above, [34]). Therefore, loss of PLCγ2 severely impacts mostly immune cells, and hampers key responses to insult. It is tempting to speculate that a reduced PLCγ2 function in brain immune cells might lead to an increased risk of dementia, however convincing data to support this are currently lacking.

At the opposite side of the spectrum, the majority of hypermorphic variants (S707Y, Y495C, D993G) have been identified in the context of APLAID disorders, and in some mouse models with autoinflammatory phenotypes [3, 109, 110]. The B-cell disturbances observed in patients with APLAID are varied, ranging from mild to severe, in line with the wide spectrum of inflammatory and immune-deficiency phenotypes. Newly characterised APLAID mutations (A708P, L848P, L845P-L848Del, M1141) have been associated with the ﻿most severe outcome described so far, including complete B-cell depletion and agamma-globulinemia, leading to severe multisystemic autoinflammation, cutaneous manifestations [100, 111], and recurrent bacterial infections [100]. Interestingly, some defects described for peripheral cells of certain patients with APLAID mutations, more closely resembled those observed in PLCγ2-deficient mice [101].

It is unknown whether innate immune cells in the brain would be similarly affected by these GOF variants, as no neurological phenotypes have been described for these patients so far. Only one study reported a patient with slight psychomotor retardation, and seizures that were attributed to CNS vasculitis [111].

Different degrees of severity have also been described for mouse models bearing hypermorphic spontaneous mutations (Ali14 and Ali5 [109, 112]). These models seem to recapitulate features of the human diseases, including chronic inflammation accompanied by hyperreactive B-cells and expansion of innate inflammatory cells [102, 112]. Again, no investigation has been carried out on the CNS, and no evident neurological phenotypes have been reported for these mice. It would be of interest to investigate whether PLCγ2 GOF can exacerbate neurodegeneration in these contexts.

Finally, genomic deletions within the *PLCG2* coding region cause PLAID disorders, which manifest with cold-induced urticaria and variable immunologic defects, including antibody deficiency and autoimmune disease [113]. PLAID-affected individuals have reduced PLCγ2-mediated signal transduction at physiological temperatures, despite constitutive enzyme activation [3, 113].

*PLCG2* GOF polymorphisms have also been recently associated with other disorders, such as inflammatory bowel disease [114] and ﻿resistance of chronic lymphocytic leukaemia cells to the BTK inhibitor ibrutinib [99, 115]. A potential link between *PLCG2* and macular degeneration has also been described [116].

From studies in the peripheral immune system, it has emerged that some of the effects of PLCγ2 GOF might be mediated via the activation of the NLRP3 inflammasome [31, 100]. Additionally, PLCγ2 activation in microglia, monocyte/macrophages, dendritic and mast cells has been linked to increased cytokine production [32, 35]. However, it remains to be demonstrated whether microglial activation of NLRP3 occurs downstream of PLCγ2 activation. Notably, the outcome of PLCγ2 GOF mutations varies greatly depending on the type of activation produced (e.g. constitutive vs stimulated) and, most importantly, on the cellular context and signalling cascade this enzyme is wired in. Indeed, in microglia, signalling through TREM2 typically dampens this pro-inflammatory response [33, 34]. The effect of PLCγ2 activation on NLRP3-mediated inflammation may be receptor dependent and, in the presence and context of TREM2 signalling, there may not be an exacerbation of this specific inflammatory response. More specific studies should be carried out to address the changes in wiring and signal transduction in microglia with PLCγ2 variants.

To the contrary, the slightly hypermorphic p.P522R polymorphism has been associated with a strong protective effect (see above). This protective action has been demonstrated with respect to neurological symptoms, and therefore it is most likely to be mediated by the brain immune cells: microglia. However, it is now known that communication between the CNS and the peripheral immune systems is crucial, and monocyte infiltration in the brain could influence microglia responses [117]. As a recent study on a centenarian cohort suggested, this variant could exert a beneficial effect on the entire immune system and promote healthy ageing [9], thus indicating that protection might also be mediated via the peripheral immune system, and could span the entire organism.

Despite the variable range of disturbances observed both in individuals and mouse/cell models with PLCγ2 mutations, too little or too much PLCγ2 activity is detrimental to immune function – as documented primarily at the peripheral level – and the effects produced by these disease-associated variants cannot be rescued by homeostatic compensatory mechanisms.

In conclusion, TREM2 signalling critically influences microglial functional and metabolic states; and PLCγ2 constitutes a central node controlling neuroinflammation both in peripheral immune cells and in brain microglia. Activation state of the TREM2/PLCγ2 pathway might be regarded as an indicator of a specific microglial responsive cell state. According to the Goldilocks principle, “just enough” activation of this pathway supports beneficial immune responses, and prevents detrimental effects impacting severely on the immune system, and its capacity to respond to insults.

### Modulation of the TREM2/PLCγ2 signalling pathways for the development of disease-modifying therapies for dementia

#### Boosting protective microglia

Although microglia are heavily implicated in the pathogenesis of AD [118], exactly how they affect AD pathology, and their interplay with Aβ and tau, are incompletely understood. Genetics provides a great starting point to the process of identifying key contributors to disease and untangling the function of key genes involved in neurodegeneration.

TREM2 is upregulated in microglia in AD human brains and in several models of neurodegenerative disorders [14]. It is unclear whether this would also lead to increased pathway activation. Key questions remain regarding how much more activation could be achieved beyond existing activation, and whether the beneficial effect could vary across disease progression. Additionally, whether positive modulation of the TREM2/PLCγ2 pathway would translate to a more efficient response to insults and clearance of dead cells, pathogenic proteins, or aggregates that accumulate with aging – as suggested in the sections above – needs further investigation. However, recent in vivo and in vitro models seem to support the idea that activation of Trem2 leads to reduced amyloidogenesis by a more efficient clearance, and promotes cell phagocytosis and survival, at least in mouse models ([46], see more below).

These protective/beneficial effects could be mediated by several cellular mechanisms:

- More efficient lipid metabolism, leading to ﻿more metabolically and immunologically responsive immune cells [19, 34]
- Increased survival [39, 119]
- Subtle activation of processes dependent on release of intracellular calcium stores – including assembly of the NLRP3 inflammasome [9], increased motility, and phagocytosis – leading to the promotion of initial immune response that stops development of aggregation-related pathology/accumulation of damaged membranes [120]

Additionally, non-cell autonomous effects on other cell types, due to microglial direct interaction or release of cytokines and neurotrophic or synaptotrophic molecules, such as TNF-alpha [121, 122], could contribute to maintain a healthy CNS throughout ageing [123].

#### Therapeutics under development

Current evidence, as described in the previous section, points to the enhancement of TREM2 signalling as a promising candidate for AD therapy [124]. Several TREM2-targeting drugs are under development, with some approaches at more advanced stages than others (reviewed in Lewcock et al. [124]). Agonistic monoclonal antibodies directed at the receptor, now in clinical trial phase 2, seem to reduce neurotoxic plaques and neurite dystrophy in a mouse model of AD [125]. Furthermore, single cell RNA-seq analysis revealed that a variant of the antibody in trial, AL002c, leads to the expansion of microglia transitioning from homeostatic to DAM, and proliferating microglia, promoting metabolic activation [125]. Other similar strategies, based on monoclonal antibodies (4D9), act via a dual mechanism: as direct agonists of the TREM2 signalling, and ﻿by stabilizing TREM2 on the cell surface and reducing its shedding [46]. This strategy demonstrated increased microglial uptake of myelin debris and amyloid β-peptide in vitro, and reduced amyloid plaque burden in the cortex of APP^NLGF^ mice [46]. Notably, Gratuze et al. [13] suggested that potentiating the TREM2 pathway might be beneficial only during a specific time window (e.g. early disease stages).

Although genetic evidence suggests that mild potentiation of PLCγ2 signalling downstream of TREM2 is protective (see above), no specific PLCγ2-targeted therapeutics have been developed to date. Additionally, the lack of a reliable inhibitor/activator directed at PLC isoforms specifically, and therefore the lack of a reference tool compound for validation, has hampered the generation of bona fide PLCγ2 modulators. Indeed, both inhibitors and activators/potentiators would provide as useful therapeutics in different diseases (from APLAID to AD, see chapter: “The spectrum of clinical phenotypes manifested by TREM2 and PLCG2 polymorphisms”). As discussed above, the newly generated structural data could help in designing ad-hoc small drug molecules that act at specific domains.

#### A note of caution

Alternative strategies could act on other enzymes in the TREM2/PLCγ2 signalling cascade. For instance, it has been suggested that p.R47H leads to hyperactivation of Akt downstream of Trem2 [38], therefore, Akt inhibitors could be considered as a potential approach to modulate disease-enhancing microglia states. However, as the main mediator of PI3K signalling, Akt is a central effector downstream of several signal transduction pathways, and it is involved in numerous cellular responses, such as protein synthesis, cell proliferation, cell motility, metabolism, survival, apoptosis, cell growth and cytokine production. Akt acts via the modulation of proteins such as mammalian target of rapamycin (mTOR), the Bcl-2 antagonist of cell death, caspase 9, glycogen synthase kinase-3 beta, p70 S6 kinase, fork head transcription factors, proline-rich Akt substrate of 40 kDa, and nuclear factor kappa B [126]. Indeed, BMDMs from Trem2-deficient mice show a defective energetic state, as a result of reduced mTOR signalling. An alternative Trem2-independent activation of mTOR ameliorates this defect [127]. Therefore, Akt inhibitors might interfere with a wide range of functions, especially the regulation of metabolic states in microglia. So far, these cascades have been mostly studied in vitro and/or in surrogate cell lines (such as BV2), and they still need to be thoroughly mapped in microglia cells and in specific disease-contexts.

As the activity of the immune system needs to be tightly balanced, immunomodulatory therapies should be evaluated with caution, in order to minimise potential side effects [128]. Microglia share much of their genetic make-up, complement of proteins, and function, with other immune cells, particularly macrophages [129]. Outside of the CNS, TREM2 and PLCγ2 are also expressed in tissue-resident macrophages. Additionally, PLCγ2 is present in circulating monocytes, platelets and B-cells, amongst other immune cells (see sections above, [3, 130]). TREM2 and PLCγ2 are important for osteoclast development and function [4]. As described above, the effects of TREM2/PLCγ2 pathway modulation are cell type dependent. Therefore, the beneficial effect of a therapeutic agent on microglia function in the brain might be accompanied by a dysregulation of other immune cells’ function and other tissues/organs, such as bones and liver [131]. Hence, it is fundamental to take into consideration the impact of TREM2/PLCγ2 modulation on the broader immune system, with special regard to conditions where the immune function is already compromised, for instance during ageing and diseases which alter normal tissue homeostasis (e.g. metabolic disorders and cancer). Given the newly identified role for the TREM2/ApoE axis in cancer [132], care should be taken when evaluating those therapies that enhance TREM2, in order to prevent unwanted effects on immunosuppressive myeloid cells [133].

## Conclusions

The last decade has seen a plethora of studies linking immune-relate genes to AD [1, 6]. These findings have placed TREM2 and PLCγ2 at the centre of mechanisms in AD pathogenesis, and as key targets for therapeutic modulation.

Despite the advances in the understanding of these pathways, and their contribution to immune cell function, many challenges remain to be addressed:

- Effects on the immune cells. It is still unknown how protection via TREM2/PLCγ2 is mediated, and whether this is due to an effect on microglia or the peripheral immune system (or an interplay of both). Further studies will also need to address how this model fits with the activation of (and crosstalk with) other immune receptors upstream of PLCγ2 in innate immune cells – including TLRs and Fc receptors.
- Role of the local environment and different cell/disease states. It would be important to determine how this pathway is regulated in different scenarios, and at different stages of disease progression – including a substrate of an ageing immune system, with altered metabolism/bioenergetics and declined immune function.
- Therapeutic considerations. We need to investigate what the best way to modulate the activity of TREM2/PLCγ2 would be, without eliciting unwanted side effects. Moreover, it will be crucial to determine at which stage of disease progression would an intervention be beneficial, and whether a genotype-based stratification of the patients would be required to identify which patients might benefit the most (e.g. based on TREM2/APOE genotype). An additional consideration for pharmacological modulation would be how tuneable the pathway is. It is still to be determined a) what the desired amount of activation is, and b) whether we can achieve a suitable amount of activation via a particular target, or modality, or mechanism.
- Risks and safety issues. Activating this pathway might affect the immune system broadly, and potentially impact on metabolism and fertility. In the CNS, this could lead to increased inflammation / synaptic pruning. In the peripheral immune system, concerns could arise around the promotion of inflammatory disorders or cancer (see above).

**Fig. 4:**
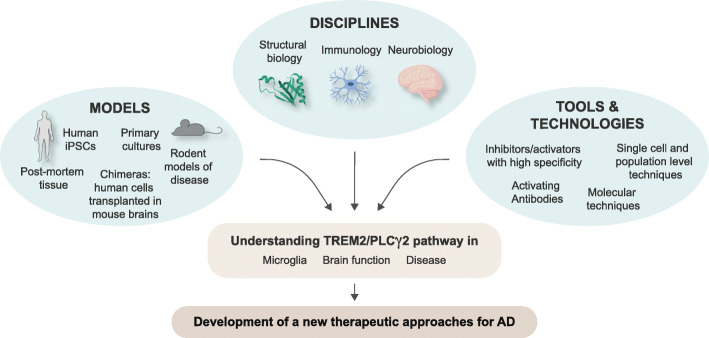
Strategies to tackle questions around TREM2/PLCγ2, and identify therapeutics for AD. The combination of efforts from several disciplines (including structural biology, immunology and neurobiology) and the assessment of mouse and human models, together with the generation of new selective pharmacological tools for TREM2/PLCγ2 will help to address these questions

The combination of efforts from several disciplines (including structural biology, immunology and neurobiology) and the assessment of mouse and human models, together with the generation of new selective pharmacological tools for TREM2/PLCγ2, will help to address these questions, and specifically modulate this pathway for protection/therapy against AD (Fig. 4). Ultimately, these findings are propelling a shift of paradigm around the role of microglia in AD: this is crucial to increase our understanding of the disease, and properly design new therapeutic strategies.

## Data Availability

The homology model of PLCγ2 is available from the authors upon request.

## Abbreviations

GWAS: Genome Wide Association Studies
TREM2: Triggering Receptor Expressed On Myeloid Cells 2
PLCγ2: Phospholipase C gamma2
LOAD: Late Onset Alzheimer’s Disease
CNS: Central Nervous System
CSF: Cerebrospinal Fluid
OR: Odds Ratio
PLAID: PLC2 Associated Antibody Deficiency
APLAID: Autoinflammation PLAID
LOF: Loss of Function
TLR: Toll Like Receptor
NLRP3: Pyrin (PYD)-domain-containing protein 3
BMDM: Bone Marrow Derived Macrophages
PI3K: Phosphoinositide 3 Kinase
KO: Knock Out
Aβ: Amyloid Beta
IPSC: Induced Pluripotent Stem Cell
DAM: Disease Associated Microglia
LDAM: Lipid Droplet Accumulating Microglia
IP1: Inositol Monophosphate
WT: Wild Type
DAP12: DNAX Activating Protein of 12 kDa
ITAM: Immunoreceptor Tyrosine Activation Motif
PM: Plasma Membrane
SYK: Spleen Tyrosine Kinase
BLNK: B-cell Linker Protein
BTK: Brutons Tyrosine Kinase
PIP3: phosphatidylinositol (3,4,5)-trisphosphate
PIP2: phosphatidylinositol (4,5)-bisphosphat
LAT: Linker for Activation of T Cells
SLP76: SH2-domain-containing leukocyte protein of 76 kDa
GADS: GRB2-related adaptor downstream of Shc
RTK: Receptor Tyrosine Kinase
spPH: Split PH
IP3: inositol (1,4,5)-trisphosphate
DAG: Diacylglycerol
PKC: Protein Kinase C
γSA: Gamma Specific Array
CRYOEM: Cryogenic Electron Microscopy
eQTL: Expression Quantitative Trait Loci
GOF: Gain of Function
TSPO: Translocator Protein
KI: Knock In
mTOR: Mammalian Target of Rapamycin

## References

[1] Sims R, van der Lee SJ, Naj AC, Bellenguez C, Badarinarayan N, Jakobsdottir J (2017). Novel rare coding variants in PLCG2, ABI3 and TREM2 implicate microglial-mediated innate immunity in Alzheimer’s disease. Nat Genet.

[2] Colonna M, Wang Y (2016). TREM2 variants: new keys to decipher Alzheimer disease pathogenesis. Nat Rev Neurosci.

[3] Koss H, Bunney TD, Behjati S, Katan M (2014). Dysfunction of phospholipase Cγ in immune disorders and cancer. Trends Biochem Sci.

[4] Xing J, Titus AR, Humphrey MB (2015). The TREM2-DAP12 signaling pathway in Nasu–Hakola disease: a molecular genetics perspective. Res Rep Biochem.

[5] Wang D, Feng J, Wen R, Marine J-C, Sangster MY, Parganas E (2000). Phospholipase Cγ2 is essential in the functions of B cell and several fc receptors. Immunity..

[6] Guerreiro R, Wojtas A, Bras J, Carrasquillo M, Rogaeva E, Majounie E (2013). TREM2 variants in Alzheimer’s disease. N Engl J Med.

[7] Yeh FL, Hansen DV, Sheng M (2017). TREM2, microglia, and neurodegenerative diseases. Trends Mol Med.

[8] Kleineidam L, Chouraki V, Próchnicki T, van der Lee SJ, Madrid-Márquez L, Wagner-Thelen H (2020). PLCG2 protective variant p.P522R modulates tau pathology and disease progression in patients with mild cognitive impairment. Acta Neuropathol.

[9] van der Lee SJ, Conway OJ, Jansen I, Carrasquillo MM, Kleineidam L, van den Akker E (2019). A nonsynonymous mutation in PLCG2 reduces the risk of Alzheimer’s disease, dementia with Lewy bodies and frontotemporal dementia, and increases the likelihood of longevity. Acta Neuropathol.

[10] de Rojas I, Moreno-Grau S, Tesi N, Grenier-Boley B, Andrade V, Jansen I, et al. Common variants in Alzheimer’s disease: Novel association of six genetic variants with AD and risk stratification by polygenic risk scores. medRxiv. 2020;19012021.

[11] Bellenguez C, Kucukali F, Jansen I, Andrade V, Morenau-Grau S, Amin N, et al. Large meta-analysis of genome-wide association studies expands knowledge of the genetic etiology of Alzheimer disease and highlights potential translational opportunities. medRxiv. 2020; Available from: http://medrxiv.org/content/early/2020/10/03/2020.10.01.20200659.abstract.

[12] Tsai AP, Dong C, Preuss C, Moutinho M, Lin PB-C, Hajicek N, et al. PLCG2 as a Risk Factor for Alzheimer’s Disease. bioRxiv. 2020;2020.05.19.104216 Available from: http://biorxiv.org/content/early/2020/05/22/2020.05.19.104216.abstract.

[13] Gratuze M, Leyns CEG, Sauerbeck AD, St-Pierre MK, Xiong M, Kim N (2020). Impact of TREM2R47H variant on tau pathology-induced gliosis and neurodegeneration. J Clin Invest.

[14] Keren-Shaul H, Spinrad A, Weiner A, Matcovitch-Natan O, Dvir-Szternfeld R, Ulland TK (2017). A Unique Microglia Type Associated with Restricting Development of Alzheimer’s Disease. Cell.

[15] McQuade A, Kang YJ, Hasselmann J, Jairaman A, Sotelo A, Coburn M (2020). Gene expression and functional deficits underlie TREM2-knockout microglia responses in human models of Alzheimer’s disease. Nat Commun.

[16] Yuan P, Condello C, Keene CD, Wang Y, Bird TD, Paul SM (2016). TREM2 Haplodeficiency in mice and humans impairs the microglia barrier function leading to decreased amyloid compaction and severe axonal dystrophy. Neuron..

[17] Yeh FL, Wang Y, Tom I, Gonzalez LC, Sheng M (2016). TREM2 binds to Apolipoproteins, including APOE and CLU/APOJ, and thereby facilitates uptake of amyloid-Beta by microglia. Neuron..

[18] Wang Y, Ulland TK, Ulrich JD, Song W, Tzaferis JA, Hole JT (2016). TREM2-mediated early microglial response limits diffusion and toxicity of amyloid plaques. J Exp Med.

[19] Nugent AA, Lin K, van Lengerich B, Lianoglou S, Przybyla L, Davis SS (2020). TREM2 regulates microglial cholesterol metabolism upon chronic phagocytic challenge. Neuron..

[20] Marschallinger J, Iram T, Zardeneta M, Lee SE, Lehallier B, Haney MS (2020). Lipid-droplet-accumulating microglia represent a dysfunctional and proinflammatory state in the aging brain. Nat Neurosci.

[21] Minhas PS, Latif-Hernandez A, McReynolds MR, Durairaj AS, Wang Q, Rubin A (2021). Restoring metabolism of myeloid cells reverses cognitive decline in ageing. Nature.

[22] Li JT, Zhang Y (2018). TREM2 regulates innate immunity in Alzheimer’s disease. J Neuroinflam.

[23] Deczkowska A, Weiner A, Amit I (2020). The physiology, pathology, and potential therapeutic applications of the TREM2 signaling pathway. Cell..

[24] Uhlén M, Fagerberg L, Hallström BM, Lindskog C, Oksvold P, Mardinoglu A (2015). Tissue-based map of the human proteome. Science..

[25] Wen R, Jou S-T, Chen Y, Hoffmeyer A, Wang D (2002). Phospholipase Cγ2 is essential for specific functions of FcεR and FcγR. J Immunol.

[26] Magno L, Lessard CB, Martins M, Lang V, Cruz P, Asi Y (2019). Alzheimer’s disease phospholipase C-gamma-2 (PLCG2) protective variant is a functional hypermorph. Alzheimers Res Ther.

[27] Jakus Z, Simon E, Frommhold D, Sperandio M, Mócsai A (2009). Critical role of phospholipase Cγ2 in integrin and fc receptor-mediated neutrophil functions and the effector phase of autoimmune arthritis. J Exp Med.

[28] Wilde JI, Watson SP (2001). Regulation of phospholipase C γ isoforms in haematopoietic cells - why one, not the other?. Cell Signal.

[29] Hashimoto A, Takeda K, Inaba M, Sekimata M, Kaisho T, Ikehara S (2000). Cutting edge: essential role of phospholipase C-γ2 in B cell development and function. J Immunol.

[30] Ito H, Hamerman JA (2012). TREM-2, triggering receptor expressed on myeloid cell-2, negatively regulates TLR responses in dendritic cells. Eur J Immunol.

[31] Chae JJ, Park YH, Park C, Hwang IY, Hoffmann P, Kehrl JH (2015). Brief report: connecting two pathways through Ca2+ signaling: NLRP3 inflammasome activation induced by a hypermorphic PLCG2 mutation. Arthritis Rheum.

[32] Aki D, Minoda Y, Yoshida H, Watanabe S, Yoshida R, Takaesu G (2008). Peptidoglycan and lipopolysaccharide activate PLCγ2, leading to enhanced cytokine production in macrophages and dendritic cells. Genes Cells.

[33] Liu W, Taso O, Wang R, Bayram S, Graham AC, Garcia-Reitboeck P (2020). Trem2 promotes anti-inflammatory responses in microglia and is suppressed under pro-inflammatory conditions. Hum Mol Genet.

[34] Andreone BJ, Przybyla L, Llapashtica C, Rana A, Davis SS, van Lengerich B (2020). Alzheimer’s-associated PLCγ2 is a signaling node required for both TREM2 function and the inflammatory response in human microglia. Nat Neurosci.

[35] Takalo M, Wittrahm R, Wefers B, Parhizkar S, Jokivarsi K, Kuulasmaa T (2020). The Alzheimer’s disease-associated protective Plcγ2-P522R variant promotes immune functions. Mol Neurodegener.

[36] Maguire E, Menzies GE, Phillips T, Sasner M, Williams HM, Czubala MA, et al. The Alzheimer’s disease protective P522R variant of PLCG2, consistently enhances stimulus-dependent PLCγ2 activation, depleting substrate and altering cell function. bioRxiv. 2020; Available from: http://biorxiv.org/content/early/2020/04/28/2020.04.27.059600.abstract.

[37] Cheng-Hathaway PJ, Reed-Geaghan EG, Jay TR, Casali BT, Bemiller SM, Puntambekar SS (2018). The Trem2 R47H variant confers loss-of-function-like phenotypes in Alzheimer’s disease. Mol Neurodegener.

[38] Sayed FA, Kodama L, Udeochu JC, Fan L, Carling GK, Le D, et al. AD-linked R47H-TREM2 mutation induces disease-enhancing proinflammatory microglial states in mice and humans. bioRxiv. 2020;2020.07.24.218719 Available from: http://biorxiv.org/content/early/2020/07/25/2020.07.24.218719.abstract.

[39] Hall-Roberts H, Agarwal D, Obst J, Smith TB, Monzón-Sandoval J, Di Daniel E (2020). TREM2 Alzheimer’s variant R47H causes similar transcriptional dysregulation to knockout, yet only subtle functional phenotypes in human iPSC-derived macrophages. Alzheimers Res Ther.

[40] Luis EO, Ortega-Cubero S, Lamet I, Razquin C, Cruchaga C, Benitez BA (2014). Frontobasal gray matter loss is associated with the TREM2 p.R47H variant. Neurobiol Aging.

[41] Slattery CF, Beck JA, Harper L, Adamson G, Abdi Z, Uphill J (2014). R47H TREM2 variant increases risk of typical early-onset Alzheimer’s disease but not of prion or frontotemporal dementia. Alzheimers Dement.

[42] Jonsson T, Stefansson H, Steinberg S, Jonsdottir I, Jonsson PV, Snaedal J (2012). Variant of TREM2 associated with the risk of Alzheimer’s disease. N Engl J Med.

[43] Soragna D, Tupler R, Ratti MT, Montalbetti L, Papi L, Sestini R (2003). An Italian family affected by Nasu-Hakola disease with a novel genetic mutation in the TREM2 gene. J Neurol Neurosurg Psychiatry.

[44] Borroni B, Ferrari F, Galimberti D, Nacmias B, Barone C, Bagnoli S (2014). Heterozygous TREM2 mutations in frontotemporal dementia. Neurobiol Aging.

[45] Thornton P, Sevalle J, Deery MJ, Fraser G, Zhou Y, Ståhl S (2017). TREM 2 shedding by cleavage at the H157-S158 bond is accelerated for the Alzheimer’s disease-associated H157Y variant. EMBO Mol Med.

[46] Schlepckow K, Monroe KM, Kleinberger G, Cantuti-Castelvetri L, Parhizkar S, Xia D (2020). Enhancing protective microglial activities with a dual function TREM 2 antibody to the stalk region. EMBO Mol Med.

[47] Jiang T, Hou J-K, Gao Q, Yu J-T, Zhou J-S, Zhang H-DZ, Y-D (2016). TREM2 p.H157Y Variant and the Risk of Alzheimer’s Disease: A Meta-Analysis Involving 14,510 Subjects. Curr Neurovasc Res.

[48] Shirotani K, Hori Y, Yoshizaki R, Higuchi E, Colonna M, Saito T (2019). Aminophospholipids are signal-transducing TREM2 ligands on apoptotic cells. Sci Rep.

[49] Hardy J (2017). Membrane damage is at the core of Alzheimer’s disease. Lancet Neurol.

[50] Wang Y, Cella M, Mallinson K, Ulrich JD, Young KL, Robinette ML (2015). TREM2 lipid sensing sustains the microglial response in an Alzheimer’s disease model. Cell..

[51] Atagi Y, Liu CC, Painter MM, Chen XF, Verbeeck C, Zheng H (2015). Apolipoprotein E is a ligand for triggering receptor expressed on myeloid cells 2 (TREM2). J Biol Chem.

[52] Kober DL, Brett TJ, Mol J, Author B (2017). TREM2-ligand interactions in health and disease. J Mol Biol.

[53] Piers TM, Cosker K, Mallach A, Johnson GT, Guerreiro R, Hardy J (2020). A locked immunometabolic switch underlies TREM2 R47H loss of function in human iPSC-derived microglia. FASEB J.

[54] Lauro C, Limatola C (2020). Metabolic Reprograming of Microglia in the Regulation of the Innate Inflammatory Response. Front Immunol.

[55] Heslegrave A, Heywood W, Paterson R, Magdalinou N, Svensson J, Johansson P (2016). Increased cerebrospinal fluid soluble TREM2 concentration in Alzheimer’s disease. Mol Neurodegener.

[56] Ulland TK, Colonna M (2018). TREM2 — a key player in microglial biology and Alzheimer disease. Nat Rev Neurol.

[57] Jaitin DA, Adlung L, Thaiss CA, Weiner A, Li B, Descamps H (2019). Lipid-associated macrophages control metabolic homeostasis in a Trem2-dependent manner. Cell.

[58] Katan M, Cockcroft S (2020). Phospholipase C families: common themes and versatility in physiology and pathology. Prog Lipid Res.

[59] Deming Y, Filipello F, Cignarella F, Cantoni C, Hsu S, Mikesell R (2019). The MS4A gene cluster is a key modulator of soluble TREM2 and Alzheimer’s disease risk. Sci Transl Med.

[60] Yang J, Fu Z, Zhang X, Xiong M, Meng L, Zhang Z (2020). TREM2 ectodomain and its soluble form in Alzheimer’s disease. J Neuroinflam..

[61] Xu X, Li H, Xu C (2020). Structural understanding of T cell receptor triggering. Cell Mol Immunol.

[62] Whittaker GC, Orr SJ, Quigley L, Hughes L, Francischetti IMB, Zhang W (2010). The linker for activation of B cells (LAB)/non-T cell activation linker (NTAL) regulates triggering receptor expressed on myeloid cells (TREM)-2 signaling and macrophage inflammatory responses independently of the linker for activation of T cells. J Biol Chem.

[63] Forabosco P, Ramasamy A, Trabzuni D, Walker R, Smith C, Bras J (2013). Insights into TREM2 biology by network analysis of human brain gene expression data. Neurobiol Aging.

[64] Berton G, Mócsai A, Lowell CA (2005). Src and Syk kinases: key regulators of phagocytic cell activation. Trends Immunol.

[65] Boggon TJ, Eck MJ (2004). Structure and regulation of Src family kinases. Oncogene..

[66] Levinson NM, Seeliger MA, Cole PA, Kuriyan J (2008). Structural basis for the recognition of c-Src by its Inactivator Csk. Cell..

[67] Cosenza-Nashat MA, Kim MO, Zhao ML, Suh HS, Lee SC (2006). CD45 isoform expression in microglia and inflammatory cells in HIV-1 encephalitis. Brain Pathol.

[68] Courtney AH, Shvets AA, Lu W, Griffante G, Mollenauer M, Horkova V (2019). CD45 functions as a signaling gatekeeper in T cells. Sci Signal.

[69] El-Hillal O, Kurosaki T, Yamamura H, Kinet J-P, Scharenberg AM (1997). Syk kinase activation by a src kinase-initiated activation loop phosphorylation chain reaction. Proc Natl Acad Sci.

[70] Rodriguez R, Matsuda M, Storey A, Katan M (2003). Requirements for distinct steps of phospholipase Cγ2 regulation, membrane-raft-dependent targeting and subsequent enzyme activation in B-cell signalling. Biochem J.

[71] Peng Q, Malhotra S, Torchia JA, Kerr WG, Coggeshall KM, Humphrey MB (2010). TREM2- and DAP12-dependent activation of PI3K requires DAP10 and is inhibited by SHIP1. Sci Signal.

[72] Manna A, Zhao H, Wada J, Balagopalan L, Tagad HD, Appella E (2018). Cooperative assembly of a four-molecule signaling complex formed upon T cell antigen receptor activation. Proc Natl Acad Sci U S A.

[73] Bunney TD, Opaleye O, Roe SM, Vatter P, Baxendale RW, Walliser C (2009). Structural insights into formation of an active signaling complex between Rac and phospholipase C gamma 2. Mol Cell.

[74] Wist M, Meier L, Gutman O, Haas J, Endres S, Zhou Y (2020). Noncatalytic Bruton’s tyrosine kinase activates PLCγ2 variants mediating ibrutinib resistance in human chronic lymphocytic leukemia cells. J Biol Chem.

[75] Bunney TD, Katan M (2010). Phosphoinositide signalling in cancer: beyond PI3K and PTEN. Nat Rev Cancer.

[76] Bunney TD, Katan M (2011). PLC regulation: emerging pictures for molecular mechanisms. Trends Biochem Sci.

[77] Griner EM, Kazanietz MG (2007). Protein kinase C and other diacylglycerol effectors in cancer. Nat Rev Cancer.

[78] Hammond GRV, Burke JE (2020). Novel roles of phosphoinositides in signaling, lipid transport, and disease. Curr Opin Cell Biol.

[79] Katan M, Cockcroft S (2020). Phosphatidylinositol (4,5) bisphosphate: diverse functions at the plasma membrane. Essays Biochem.

[80] Huang J, Liu CH, Hughes SA, Postma M, Schwiening CJ, Hardie RC (2010). Activation of TRP channels by protons and Phosphoinositide depletion in Drosophila photoreceptors. Curr Biol.

[81] Molinari G (2015). Is hydrogen ion (H+) the real second messenger in calcium signalling?. Cell Signal.

[82] Gratuze M, Leyns CEG, Holtzman DM (2018). New insights into the role of TREM2 in Alzheimer’s disease. Mol Neurodegener.

[83] Hajicek N, Keith NC, Siraliev-Perez E, Temple BRS, Huang W, Zhang Q (2019). Structural basis for the activation of plc-γ isozymes by phosphorylation and cancer-associated mutations. Elife..

[84] Liu Y, Bunney TD, Khosa S, Macé K, Beckenbauer K, Askwith T (2020). Structural insights and activating mutations in diverse pathologies define mechanisms of deregulation for phospholipase C gamma enzymes. EBioMedicine..

[85] Jacobson MP, Friesner RA, Xiang Z, Honig B (2002). On the role of the crystal environment in determining protein side-chain conformations. J Mol Biol.

[86] Jacobson MP, Pincus DL, Rapp CS, Day TJF, Honig B, Shaw DE (2004). A hierarchical approach to all-atom protein loop prediction. Proteins Struct Funct Genet.

[87] Bunney TD, Esposito D, Mas-Droux C, Lamber E, Baxendale RW, Martins M (2012). Structural and functional integration of the PLCγ interaction domains critical for regulatory mechanisms and signaling deregulation. Structure..

[88] Bae JH, Lew ED, Yuzawa S, Tomé F, Lax I, Schlessinger J (2009). The selectivity of receptor tyrosine kinase signaling is controlled by a secondary SH2 domain binding site. Cell..

[89] Ishiai M, Sugawara H, Kurosaki M, Kurosaki T (1999). Cutting edge: association of phospholipase C-γ2 Src homology 2 domains with BLNK is critical for B cell antigen receptor signaling. J Immunol.

[90] Bae YS, Lee HY, Jung YS, Lee M, Suh PG (2017). Phospholipase Cγ in toll-like receptor-mediated inflammation and innate immunity. Adv Biol Regul.

[91] Sierksma A, Lu A, Mancuso R, Fattorelli N, Thrupp N, Salta E (2020). Novel Alzheimer risk genes determine the microglia response to amyloid-β but not to TAU pathology. EMBO Mol Med.

[92] Efthymiou AG, Goate AM (2017). Late onset Alzheimer’s disease genetics implicates microglial pathways in disease risk. Mol Neurodegener.

[93] McQuade A, Blurton-Jones M (2019). Microglia in Alzheimer’s disease: exploring how genetics and phenotype influence risk. J Mol Biol.

[94] Zheng H, Cheng B, Li Y, Li X, Chen X, Zhang Y (2018). TREM2 in Alzheimer’s disease: microglial survival and energy metabolism. Front Aging Neurosci.

[95] Paloneva J, Manninen T, Christman G, Hovanes K, Mandelin J, Adolfsson R (2002). Mutations in two genes encoding different subunits of a receptor signaling complex result in an identical disease phenotype. Am J Hum Genet.

[96] Xiang X, Piers TM, Wefers B, Zhu K, Mallach A, Brunner B (2018). The Trem2 R47H Alzheimer’s risk variant impairs splicing and reduces Trem2 mRNA and protein in mice but not in humans. Mol Neurodegener.

[97] Song W, Hooli B, Mullin K, Jin SC, Cella M, Ulland TK (2017). Alzheimer’s disease-associated TREM2 variants exhibit either decreased or increased ligand-dependent activation. Alzheimers Dement.

[98] Kleinberger G, Yamanishi Y, Suárez-Calvet M, Czirr E, Lohmann E, Cuyvers E (2014). TREM2 mutations implicated in neurodegeneration impair cell surface transport and phagocytosis. Sci Transl Med.

[99] Liu T-M, Woyach JA, Zhong Y, Lozanski A, Lozanski G, Dong S (2015). Hypermorphic mutation of phospholipase C, gamma 2 acquired in ibrutinib resistant CLL confers BTK independency upon BCR activation. Blood..

[100] Martín-Nalda A, Fortuny C, Rey L, Bunney TD, Alsina L, Esteve-Solé A (2020). Severe autoinflammatory manifestations and antibody deficiency due to novel Hypermorphic PLCG2 mutations. J Clin Immunol.

[101] Novice T, Kariminia A, Del Bel KL, Lu H, Sharma M, Lim CJ (2020). A Germline mutation in the C2 domain of PLCγ2 associated with gain-of-function expands the phenotype for PLCG2-related diseases. J Clin Immunol.

[102] Zhou Q, Lee GS, Brady J, Datta S, Katan M, Sheikh A (2012). A hypermorphic missense mutation in PLCG2, encoding phospholipase Cγ2, causes a dominantly inherited autoinflammatory disease with immunodeficiency. Am J Hum Genet.

[103] Nizami S, Hall-Roberts H, Warrier S, Cowley SA, Di Daniel E (2019). Microglial inflammation and phagocytosis in Alzheimer’s disease: potential therapeutic targets. Br J Pharmacol.

[104] Ewers M, Franzmeier N, Suárez-Calvet M, Morenas-Rodriguez E, Caballero MAA, Kleinberger G (2019). Increased soluble TREM2 in cerebrospinal fluid is associated with reduced cognitive and clinical decline in Alzheimer’s disease. Sci Transl Med.

[105] Deczkowska A, Keren-Shaul H, Weiner A, Colonna M, Schwartz M, Amit I (2018). Disease-associated microglia: a universal immune sensor of Neurodegeneration. Cell..

[106] Ford JW, McVicar DW (2009). TREM and TREM-like receptors in inflammation and disease. Curr Opin Immunol.

[107] Kang SS, Kurti A, Baker KE, Liu CC, Colonna M, Ulrich JD (2018). Behavioral and transcriptomic analysis of Trem2-null mice: not all knockout mice are created equal. Hum Mol Genet.

[108] Lee CYD, Daggett A, Gu X, Jiang L-L, Langfelder P, Li X (2018). Elevated TREM2 Gene Dosage Reprograms Microglia Responsivity and Ameliorates Pathological Phenotypes in Alzheimer’s Disease Models. Neuron.

[109] Abe K, Fuchs H, Boersma A, Hans W, Yu P, Kalaydjiev S (2011). A novel N-ethyl-N-nitrosourea-induced mutation in phospholipase Cγ2 causes inflammatory arthritis, metabolic defects, and male infertility in vitro in a murine model. Arthritis Rheum.

[110] Everett KI, Bunney TD, Yoon Y, Rodrigues-Lima F, Harris R, Driscoll PC (2009). Characterization of phospholipase Cγ enzymes with gain-of-function mutations. J Biol Chem.

[111] Morán-Villaseñor E, Saez-de-Ocariz M, Torrelo A, Arostegui JI, Yamazaki-Nakashimada MA, Alcántara-Ortigoza MA (2019). Expanding the clinical features of autoinflammation and phospholipase Cγ2-associated antibody deficiency and immune dysregulation by description of a novel patient. J Eur Acad Dermatology Venereol.

[112] Yu P, Constien R, Dear N, Katan M, Hanke P, Bunney TD (2005). Autoimmunity and inflammation due to a gain-of-function mutation in phospholipase Cγ2 that specifically increases external Ca2+ entry. Immunity..

[113] Ombrello MJ, Remmers EF, Sun G, Freeman AF, Datta S, Torabi-Parizi P (2012). Cold Urticaria, immunodeficiency, and autoimmunity related to PLCG2 deletions. N Engl J Med.

[114] de Lange KM, Moutsianas L, Lee JC, Lamb CA, Luo Y, Kennedy NA (2017). Genome-wide association study implicates immune activation of multiple integrin genes in inflammatory bowel disease. Nat Genet.

[115] Walliser C, Wist M, Hermkes E, Zhou Y, Schade A, Haas J (2018). Functional characterization of phospholipase C-γ_2_ mutant protein causing both somatic ibrutinib resistance and a germline monogenic autoinflammatory disorder. Oncotarget..

[116] Waksmunski AR, Grunin M, Kinzy TG, Igo RP, Haines JL, Bailey JNC (2019). Pathway analysis integrating genome-wide and functional data identifies PLCG2 as a candidate gene for age-related macular degeneration. Investig Ophthalmol Vis Sci.

[117] Dionisio-Santos DA, Olschowka JA, O’Banion MK (2019). Exploiting microglial and peripheral immune cell crosstalk to treat Alzheimer’s disease. J Neuroinflam.

[118] Hansen DV, Hanson JE, Sheng M (2018). Microglia in Alzheimer’s disease. J Cell Biol.

[119] Zheng H, Jia L, Liu C-C, Rong Z, Zhong L, Yang L (2017). TREM2 promotes microglial survival by activating Wnt/β-catenin pathway. J Neurosci.

[120] Conway OJ, Carrasquillo MM, Wang X, Bredenberg JM, Reddy JS, Strickland SL (2018). ABI3 and PLCG2 missense variants as risk factors for neurodegenerative diseases in Caucasians and African Americans. Mol Neurodegener.

[121] Ren S, Yao W, Tambini MD, Yin T, Norris KA, D’adamio L (2020). Microglia TREM2R47H alzheimer-linked variant enhances excitatory transmission and reduces LTP via increased TNF-α levels. Elife..

[122] York EM, Bernier LP, MacVicar BA (2018). Microglial modulation of neuronal activity in the healthy brain. Dev Neurobiol.

[123] Srinivasan K, Friedman BA, Etxeberria A, Huntley MA, van der Brug MP, Foreman O (2020). Alzheimer’s patient microglia exhibit enhanced aging and unique transcriptional activation. Cell Rep.

[124] Lewcock JW, Schlepckow K, Di Paolo G, Tahirovic S, Monroe KM, Haass C (2020). Emerging Microglia Biology Defines Novel Therapeutic Approaches for Alzheimer’s Disease. Neuron..

[125] Wang S, Mustafa M, Yuede CM, Salazar SV, Kong P, Long H, et al. Anti-human TREM2 induces microglia proliferation and reduces pathology in an Alzheimer’s disease model. J Exp Med. 2020;217. Available from. 10.1084/jem.20200785. 10.1084/jem.20200785 PMC7478730 32579671

[126] Cianciulli A, Porro C, Calvello R, Trotta T, Lofrumento DD, Panaro MA (2020). Microglia mediated neuroinflammation: focus on PI3K modulation. Biomolecules..

[127] Ulland TK, Song WM, Huang SC-C, Ulrich JD, Sergushichev A, Beatty WL (2017). TREM2 Maintains Microglial Metabolic Fitness in Alzheimer’s Disease. Cell.

[128] Golde TE (2019). Harnessing Immunoproteostasis to treat neurodegenerative disorders. Neuron..

[129] Ginhoux F, Greter M, Leboeuf M, Nandi S, See P, Gokhan S (2010). Fate Mapping Analysis Reveals That Adult Microglia Derive from Primitive Macrophages. Science..

[130] Deczkowska A, Amit I, Schwartz M (2018). Microglial immune checkpoint mechanisms. Nat Neurosci.

[131] Martin-Estebane M, Gomez-Nicola D (2020). Targeting microglial population dynamics in Alzheimer’s disease: are we ready for a potential impact on immune function?. Front Cell Neurosci.

[132] Katzenelenbogen Y, Sheban F, Katzenelenbogen Y, Sheban F, Yalin A, Yofe I (2020). Coupled scRNA-Seq and intracellular protein activity reveal an immunosuppressive role of TREM2 in Cancer. Cell..

[133] Bugler-Lamb A, Guilliams M (2020). Myeloid cells TREM down anti-tumor responses. Cell..

